# The use of aromatase inhibitors in boys with short stature: what to know before prescribing?

**DOI:** 10.1590/2359-3997000000284

**Published:** 2017-07-01

**Authors:** Alessandra Linardi, Durval Damiani, Carlos A. Longui

**Affiliations:** 1 Departamento de Fisiologia Faculdade de Ciências Médicas Santa Casa de São Paulo São Paulo SP Brasil Departamento de Fisiologia, Unidade de Farmacologia, Faculdade de Ciências Médicas da Santa Casa de São Paulo (FCMSCSP), São Paulo, SP, Brasil; 2 Hospital das Clínicas Faculdade de Medicina Universidade de São Paulo São Paulo SP Brasil Departamento de Pediatria, Unidade de Endocrinologia Pediátrica, Hospital das Clínicas da Faculdade de Medicina da Universidade de São Paulo (HCFMUSP), São Paulo, SP, Brasil; 3 Departamento de Fisiologia Unidade de Endocrinologia Pediátrica FCMSCSP São Paulo SP Brasil Departamento de Fisiologia, Disciplina de Medicina Molecular, Unidade de Endocrinologia Pediátrica, FCMSCSP, São Paulo, SP, Brasil

**Keywords:** Anastrozole, letrozole, short stature, drug interaction, cytochrome P450

## Abstract

Aromatase is a cytochrome P450 enzyme (CYP19A1 isoform) able to catalyze the conversion of androgens to estrogens. The aromatase gene mutations highlighted the action of estrogen as one of the main regulators of bone maturation and closure of bone plate. The use of aromatase inhibitors (AI) in boys with short stature has showed its capability to improve the predicted final height. Anastrozole (ANZ) and letrozole (LTZ) are nonsteroidal inhibitors able to bind reversibly to the heme group of cytochrome P450. In this review, we describe the pharmacokinetic profile of both drugs, discussing possible drug interactions between ANZ and LTZ with other drugs. AIs are triazolic compounds that can induce or suppress cytochrome P450 enzymes, interfering with metabolism of other compounds. Hydroxilation, N-dealkylation and glucoronidation are involved in the metabolism of AIs. Drug interactions can occur with azole antifungals, such as ketoconazole, by inhibiting CYP3A4 and by reducing the clearance of AIs. Antiepileptic drugs (lamotrigine, phenobarbital, and phenytoin) also inhibit aromatase. Concomitant use of phenobarbital or valproate has a synergistic effect on aromatase inhibition. Therefore, it is important to understand the pharmacokinetics of AIs, recognizing and avoiding possible drug interactions and offering a safer prescription profile of this class of aromatase inhibitors. Arch Endocrinol Metab. 2017;61(3):391-7.

## INTRODUCTION

Aromatase is a cytochrome P450 enzyme, identified as the CYP19A1 isoform, which catalyzes the conversion of androstenedione and testosterone to estrone and estradiol, respectively. The enzyme is a complex formed by two proteins: CYP19 and nicotinamide-adenine dinucleotide phosphate reductase (NADPH) (1).

In males, androgens are produced both by the adrenal glands and the testes, whereas estrogen is mostly synthesized locally in peripheral tissues from the local aromatization of circulating androgens (2,3). Adipose tissue is the primary site of aromatization, but aromatase can be found at other sites such as the brain (hypothalamus, limbic system, and cerebral cortex), breast, placenta, liver, muscle, bone (osteoblast and chondrocyte), testes (Leydig cell and germ cell), vasculature (smooth muscle cell), and skin (fibroblast and hair follicle) (4,5). Peripherally synthesized estrogen seems to have predominantly local effects (2,3,6).

Men with congenital aromatase deficiency have tall stature associated with delayed fusion of the epiphyseal growth plate, osteoporosis, overweight, glucose intolerance, hyperlipidemia, and reduction of fertility. In girls, virilization of the external genitalia can result in atypical genitalia. Hypogonadism can also occur, with inappropriate mammary gland development and primary amenorrhea (7-9). On the other hand, aromatase excess syndrome is caused by sub chromosomal recombination of *CYP19A1* gene (duplication, deletion, and inversion), with concomitant recruitment of new promoters. The clinical presentation includes gynecomastia and bone age advancement with potential reduction of final height (10).

Estrogen, as well as its receptors, plays a key role in bone maturation (11). The use of aromatase inhibitors, with the goal of reducing the estrogen-dependent skeletal maturation rate has been proposed in boys receiving treatment for short stature (12,13). The effects of these drugs upon growth appear to be mediated by the reduction of estrogen concentration at the epiphyseal chondrocyte level, associated with decreased circulating estrogen concentration and consequent reduction in the secretion of both growth hormone (GH) and insulin-like o growth factor 1 (IGF-1) (14,15). Estrogen has apparently small relevance in the regulation of growth in the prepubertal period, but even at low concentration increases growth during puberty, as well as at high concentration determines maturation and closure of the epiphyses at the final stage of puberty (16).

Aromatase inhibitors can be divided into type I and type II. Type I inhibitors are steroidal and derivatives of androstenedione, the natural substrate of the aromatase enzyme. They bind irreversibly to the active site, thereby permanently inactivating the enzyme. The inhibitors of type II are nonsteroidal and bind to the heme group of cytochrome P450. Despite the strong binding, these are reversible inhibitors. The third-generation nonsteroidal aromatase inhibitors, anastrozole and letrozole, are the most effective blocking compounds (> 95%) by suppressing the formation of the estrogens estrone (E1) and estradiol (E2) (17). Additionally, type II inhibitors have a longer half-life, which allows for a single daily administration. Due to the higher potency of letrozole, a greater increase in the concentrations of gonadotropins and testosterone has been reported in boys treated with letrozole when compared to the group treated with anastrozole (18).

Considering the increased prescription of both aromatase inhibitors in adolescents with short stature, the aim of this review is to discuss the possible drug interactions between anastrozole and letrozole and other drugs. In addition, a brief description of the pharmacokinetic profile of both inhibitors was included in this review in order to understand the emergence of possible drug interactions related to absorption, metabolism, half-life and excretion of these drugs.

## PHARMACOKINETICS

Anastrozole and letrozole are administered once daily orally at doses of 1 mg and 2.5 mg, respectively. Maximum estradiol suppression is reached around 2 to 4 days for them both. Oral absorption of both inhibitors is not significantly affected by food (18).

Anastrozole reaches the equilibrium plasma concentration, also known as steady state*,* after 7 days, while it takes letrozole around 45-60 days. This fact is due to the probable nonlinear kinetics of letrozole. The half-life of anastrozole after a single 1-mg dose is 48 hours, while that of letrozole is 4 days or more after a 2.5-mg oral dose (19). At therapeutic doses, around 40% of the anastrozole is bound to plasma proteins (20), while this protein binding rate is around 60% for letrozole (21).

Anastrozole and letrozole are triazolic derivatives and therefore have a nitrogen-containing heterocyclic ring. Many azole compounds can be inhibitors or inducers of cytochrome P450 and, consequently, interfere with the metabolism of other substances depending on the monoamino oxigenase system. Lipophilic compounds containing heterocyclic nitrogen, such as phenylpyridines and phenylimidazoles, fit this profile (22,23).

**Figure 1:**
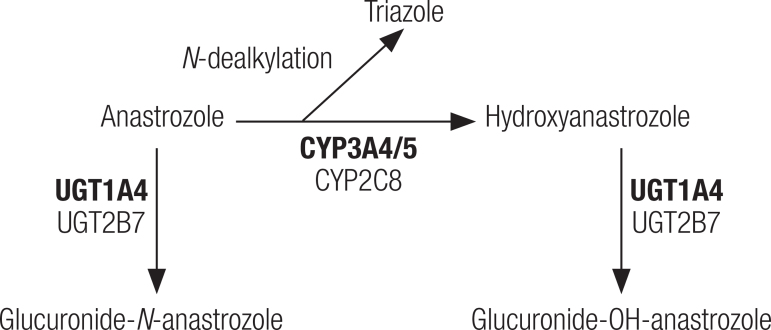
Anastrozole metabolic pathways. Anastrozole can be coupled directly to glucuronide-N-anastrozole or previously converted to hydroxyanastrozole via cytochrome P450 (CYPs) and subsequent conjugation to glucuronide-OH-anastrozole. In addition, anastrozole can undergo dealkylation by CYP3A4, thereby releasing the triazole ring. The main isoforms involved in anastrozole metabolic pathways are in bold. CYP3A4, CYP3A5, and CYP2C8: cytochrome P450 isoforms. UGT1A4 and UGT2B7: uridine glucuronyl transferase (UGT) isoforms.

Anastrozole, via cytochrome P450 (CYP), is metabolized into hydroxyanastrozole or can also undergo *N*-dealkylation. The isoforms involved were identified mainly as CYP3A4 and, less importantly, CYP3A5 and CYP2C8. Subsequently, hydroxyanastrozole is extensively conjugated by glucuronidation (Figure 1). The main enzyme involved in this step is uridine glucuronyl transferase 1A4 (UGT1A4). UGT1A3 and UGT2B7 can also act in this phase, as a secondary pathway (24,25). Anastrozole can also undergo direct *in vitro* conjugation (24) without prior involvement of cytochrome P450 enzymes. In addition, anastrozole is also able to inhibit, *in vitro*, CYP1A2, 2C9, and 3A4 in human microsomes*.* However, this inhibition occurs to a much lesser extent than that described for aromatase, and the concentrations required for this purpose are not reached in the systemic circulation with the use of therapeutic doses (23).

**Figure 2:**
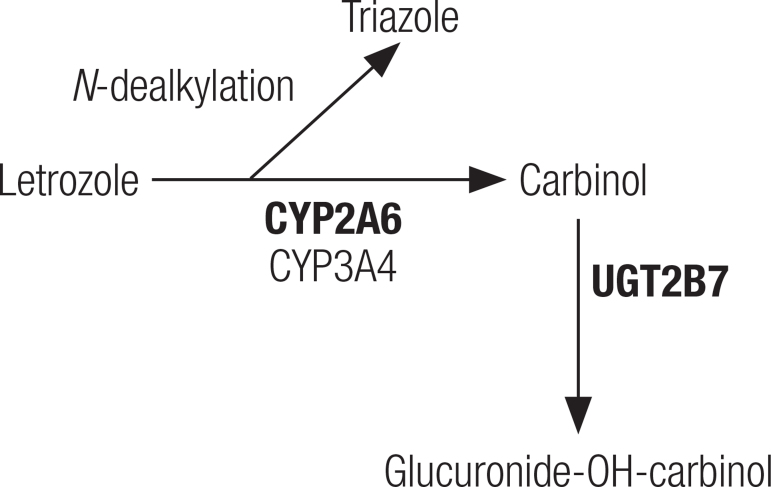
Letrozole metabolic pathways. Letrozole is converted previously to carbinol via cytochrome P450 (CYPs) and then, subsequently, the carbinol is conjugated to a glucuronide. Letrozole can undergo dealkylation, thus releasing the triazole ring. The main isoforms involved in the metabolic pathways are in bold. CYP2A6 and CYP3A4: cytochrome P450 isoforms. UGT2B7: uridine glucuronyl transferase (UGT) isoform.

Letrozole is also cleared via hepatic metabolism. *In vitro* studies have demonstrated that CYP3A4 and CYP2A6 are involved in the metabolism of letrozole, converting it to carbinol (25,26). Carbinol is then conjugated by glucuronyl transferase, more precisely by the UGT2B7 isoform (Figure 2) (27). The drug has a high affinity for CYP2A6 and a low affinity for CYP3A4. Although letrozole is metabolized by CYP2A6, letrozole itself can exert significant inhibition over this isoform (28). When this occurs, the metabolic pathway is diverted to CYP3A4 (29). This phenomenon seems to explain why letrozole shows nonlinear kinetics. In women with breast cancer following repeated doses of letrozole, there was observed a 28% increase in the area under the curve and a 42% increase in the drug’s half-life when comparing such pharmacokinetic parameters to those obtained from a single administration. Furthermore, the maximum letrozole plasma concentration was 107 nmol/L after a single dose and 467 nmol/L after repeated doses (28).

## DRUG INTERACTIONS

Azole antifungals, commonly used in clinical practice, can interfere with the activity of aromatase (30). The maximum plasma concentrations of oral and topical azole formulations and the IC50 of aromatase were determined after using testosterone as a substrate. Comparison with letrozole inhibitory potency was then performed. Among the azoles studied, miconazole (oral) and bifonazole (topical) were those that approached letrozole in its ability to inhibit aromatase. The other azoles studied were: clotrimazole (topical), itraconazole (oral), ketoconazole (oral) and miconazole (vaginal). They also exerted an inhibitory effect upon aromatase, but on a much smaller scale. Importantly, this ratio takes the maximum plasma concentrations achieved by antifungals into consideration, which means that an increase in drug absorption can induce greater inhibition of aromatase. Furthermore, there is the possibility of inhibition of CYP3A4, especially by ketoconazole**,** which reduces the clearance of letrozole and anastrozole when used concurrently with ketoconazole (31). Additionally, a study based on enzyme assays showed that some antiepileptic drugs can inhibit aromatase, including lamotrigine, oxycarbamazepine, tiagabine, phenobarbital, phenytoin, and ethosuximide (32). Therefore, it is important to consider that, in clinical practice, treatment with antiepileptics occurs chronically, and that, in some cases, there may be an association between the drugs. Among these, the concomitant use of phenobarbital or valproate has a synergistic effect on the inhibition of aromatase.

Regarding to the use of *Cannabis*, when studying mouse testis microsomes, it was reported that the extract of *Cannabis sativa* is capable of inhibiting the activity of CYP17A1 (P450c17), the enzyme responsible for the 17α-hydroxylation of progesterone and the subsequent formation of androstenedione, one of the substrates of aromatase (33). The authors also observed a similar result with the main constituents of *Cannabis sativa*, such as tetrahydrocannabinol (THC), cannabidiol (CBD), and cannabinol (CBN).

Alcohol consumption could induce an increase in aromatase expression in the adipose tissue of rats (34). Previously, the authors had also described an increased aromatase activity in the hippocampus of rats, induced by both 13% alcohol and red wine (35). In cultured MCF-7 breast cancer cells, there was observed an increase in the expression of aromatase following incubation with ethanol (36). On the other hand, a study conducted with women around 35 years of age showed that red wine consumption during one month induced an increase in plasma testosterone and a decrease in estrogens. The authors suggest that red wine could be a natural inhibitor of aromatase, due to the presence of chemical compounds such as the flavones and isoflavones. In this case, it is the chemicals present in red wine that predominate, rather than alcohol itself (37).

Considered together, these data seem to demonstrate that drugs such as azoles and antiepileptics may potentiate the action of aromatase inhibitors, further inhibiting the enzyme activity. In addition, *Cannabis sativa* and its correlates can reduce the formation of the substrate androstenedione and also potentiate the action of aromatase inhibitors. On the other hand, alcohol consumption can increase aromatase activity or expression.

Relative to the pharmacokinetic profile of anastrozole and letrozole, genetic polymorphisms may induce differences in the metabolic profile of aromatase inhibitors, primarily identified for letrozole. The CYP2A6 and CYP3A isoforms may have their activity increased or decreased because of polymorphisms. With respect to CYP2A6, intermediate and slow metabolizers exhibit higher letrozole plasma concentrations (38). One of the polymorphisms involving CYP2A6 is related to the genetic variation across different ethnic populations. The percentage of slow metabolizers in the Asian population is larger (10-20%) when compared to Caucasians (1.2%) (39). In addition, the CYP2A6 activity in women is greater than in men. Polymorphisms involving CYP3A4 also have relevant clinical significance. The most common one induces an increase in enzyme activity and has a higher prevalence among black (66.7%) as compared to white subjects (4.2%) (40). However, the clinical impact of genetic variants involving CYP3A4 on plasma concentrations of letrozole or anastrozole needs additional studies. Glucuronyltransferases may also have their activity altered due to polymorphisms.

Significant individual variations in the conjugation profile, involving glucuronyltransferase isoforms such as UGT1A4 and UGT2B7 are respectively involved in the metabolism of anastrozole and letrozole (41-43). Glucuronidation profile by UGT1A4 was correlated to therapeutic efficacy of anastrozole in women with breast cancer (44). Nevertheless, the clinical significance of individual or racial variability to the conjugation profile of aromatase inhibitors has not been entirely clarified, and neither has the influence on the therapeutic effect and excretion of these drugs. These aspects have not been adequately investigated in the treatment of children with a short stature.

By using human microsomes incubated in the presence of anastrozole and specific substrates for CYP1A2 (phenacetin), CYP2A6 (coumarin), CYP2C9 (tolbutamide), CYP2D6 (dextromethorphan), and CYP3A4 (nifedipine), only CYP1A2, CYP2C9 and CYP3A were shown to be inhibited by anastrozole. Plasma concentrations achieved by the chronic administration of therapeutic doses of anastrozole are 30-fold lower than those required for *in vitro* inhibition (23). Additionally, the administration of anastrozole in healthy adult volunteers did not interfere with the pharmacokinetics or pharmacodynamics of warfarin, an anticoagulant metabolized by both CYP3A4 (*R*-warfarin) and CYP2C9 (*S*-warfarin) (45).

The American database Micromedex reported only one drug interaction of moderate severity. It is important to emphasize that Micromedex classifies drug interactions as: major, when they represent a danger to life and require immediate medical intervention; moderate, when they may result in an exacerbation of the health condition and the need for a change in drug therapy; minor, which suggest a change in the clinical picture, but does not require a change in treatment; and the contraindicated, when it implies that the concomitant use of the drugs is not recommended. The moderate drug interaction with anastrozole refers to the concomitant use with tamoxifen (nonsteroidal estrogenic antagonist). In women with breast cancer, tamoxifen reduced the anastrozole plasma concentration by approximately 27% (46).

With regard to letrozole, Micromedex data show five drug interactions of major severity and three moderate interactions. Relative to the major severity interactions, we have the antineoplastic agent tegafur, in which inhibition of CYP2A6 by letrozole may result in lesser conversion of tegafur to 5-fluouracil and lower drug efficacy. In addition, the inhibition of CYP2C19 by letrozole may induce an increase in cilostazol plasma concentration, a platelet antiaggregant and vasodilator. The other three major severity interactions refer to the inhibition of CYP3A by clarithromycin (macrolide antimicrobial agent), ceritinib and idelalisib (antineoplastic agents), which results in an increase in letrozole plasma concentration. The three moderate severity interactions include: association with tamoxifen, which induces a reduction in plasma letrozole concentration; concomitant use with propranolol, during which the inhibition of CYP2C19 by letrozole may increase the concentration of propranolol; the association with ketoconazole, a potent inhibitor of CYP3A4, which can result in an increase in the concentration of letrozole. A study of postmenopausal women with breast cancer showed that tamoxifen reduces letrozole plasma concentration by 38% (47).

In human microsomes, letrozole significantly inhibits CYP2A6 and modestly CYP2C19. Carbinol, a metabolite of letrozole, has a less significant inhibitory effect on CYP2B6 and CYP2C19. Other isoforms studied were not altered by incubation with letrozole or carbinol (48).

Another relevant aspect is to consider letrozole plasma concentrations achieved during the chronic administration of the drug. The *in vitro* letrozole concentration that inhibits CYP2C19 is 40-fold greater than that observed in the *in vivo* steady state. For CYP2A6, such increase is fivefold.

Letrozole may also inhibit its own metabolism due to its greater affinity for CYP2A6. This phenomenon is responsible for higher plasma concentrations of the drug and greater individual variability. In relation to the carbinol metabolite, although a modest inhibitory effect on CYP2B6 and CYP2C19 has been observed *in vitro*, carbinol is rapidly conjugated *in vivo* by glucuronidation and subsequently excreted by the kidney (28).

Therefore, in respect of letrozole, the following considerations are important, namely: I) the nonlinear kinetics of the drug can cause significant variations in its plasma concentration and, thus, drug interactions involving CYP2A6 cannot be discarded; II) with respect to CYP2C19, the drug does not appear to provoke a significant change in the activity of this isoform of cytochrome P450.

Considering the cytochrome P450 isoforms involved in the metabolism of anastrozole and letrozole, it is important to note that polymorphisms leading to changes in the activity of CYP2A6 and CYP3A4, as well as drugs, food and herbal essentials can also act as enzyme inducers or inhibitors (Table 1). Therefore, with regard to CYP3A4, antidepressants (fluoxetine, sertraline, paroxetine, venlafaxine), antifungals (ketoconazole, itraconazole, fluconazole, voriconazole), antimicrobials (clarithromycin, erythromycin, chloramphenicol, and isoniazid), antiarrhythmics (diltiazem, verapamil, and amiodarone), protease inhibitors (indinavir, ritonavir, and saquinavir), cimetidine and nicardipine, besides grapefruit, may all inhibit CYP3A4 and reduce the metabolism of anastrozole by this isoform. On the other hand, antiepileptic drugs (carbamazepine, phenobarbital, and phenytoin), dexamethasone, and rifampicin, in addition to St. John’s wort can all induce CYP3A4 and increase the metabolism of anastrozole (49-51). When considering CYP2A6, drugs such as valproic acid, selegiline and isoniazid may inhibit the activity of this isoform. On the other hand, carbamazepine, phenobarbital, phenytoin, and clonazepam may induce CYP2A6 (50,52). In addition, minor alkaloids present in tobacco can also inhibit CYP2A6 (53,54). Therefore, inducers and inhibitors of CYP2A6 could influence the pharmacokinetic profile of letrozole.

**Table 1:** Main metabolism enzymes and potential drug interactions associated with inducers and inhibitors of CYP

Drugs	Metabolism		CYP	
	Phase I	Phase II	Inducers	Inhibitors
Anastrozole	CYP3A4	UGT1A4	CYP3A4: Carbamazepine, phenobarbital, phenytoin, dexamethasone, rifampicin, St. John’s wort	CYP3A4: Fluoxetine, sertraline, paroxetine, venlafaxine, ketoconazole, itraconazole, fluconazole, voriconazole, clarithromycin, erythromycin, chloramphenicol, isoniazid, diltiazem, verapamil, amiodarone, indinavir, saquinavir, cimetidine, nicardipine, grapefruit
Letrozole	CYP2A6 CYP3A4	UGT2B7	UGT2B7, phenytoin, and clonazepam	CYP2A6: Valproic acid, selegiline, isoniazid, letrozole, and tobacco alkaloids

Phase I: metabolism involving cytochrome P450 enzymes (CYP). Phase II: metabolism involving conjugation enzymes. CYP2A6 and CYP3A4: cytochrome P450 isoforms. UGT1A4 and UGT2B7: uridine glucuronyl transferase (UGT) isoforms. The metabolism of letrozole by CYP2A6 is saturable, given that the drug can inhibit this isoform.

Therefore, when using aromatase inhibitors we should consider the concomitance of clinically relevant interfering aspects, such as drug interaction, food and beverage ingestion, use of illicit drugs and plant extracts. Individual metabolic variability dependent on genetic background is also a clinically relevant aspect to be considered, with potential impact on the final drug effects. With this information, it is possible to select the AI with the profile that fits better to individual conditions.
